# Microstructural Changes in the Striatum and Their Impact on Motor and Neuropsychological Performance in Patients with Multiple Sclerosis

**DOI:** 10.1371/journal.pone.0101199

**Published:** 2014-07-21

**Authors:** Michele Cavallari, Antonia Ceccarelli, Guang-Yi Wang, Nicola Moscufo, Salem Hannoun, Christina R. Matulis, Jonathan S. Jackson, Bonnie I. Glanz, Rohit Bakshi, Mohit Neema, Charles R. G. Guttmann

**Affiliations:** 1 Center for Neurological Imaging, Department of Radiology, Brigham and Women's Hospital, Harvard Medical School, Boston, Massachusetts, United States of America; 2 Dipartimento di Neuroscienze, Salute Mentale e Organi di Senso (NESMOS), Università La Sapienza, Rome, Italy; 3 Laboratory for Neuroimaging Research, Department of Neurology, Harvard Medical School, Partners Multiple Sclerosis Center, Brigham and Women's Hospital, Boston, Massachusetts, United States of America; 4 Department of Radiology, Guangdong General Hospital, Guangzhou, Guangdong Province, People's Republic of China; 5 Centre de Recherche en Acquisition en Traitement de l'Image pour la Santé (CREATIS), Centre National de la Recherche Scientifique (CNRS) Unités Mixtes de Recherche (UMR) 5220 & Institut National de la Santé et de la Recherche Médicale (INSERM) U1044, Université Claude Bernard - Lyon 1, University of Lyon, Lyon, France; 6 Observatoire Français de la Sclérose en Plaques (OFSEP), University of Lyon, Lyon, France; Centre Hospitalier Universitaire Vaudois Lausanne - CHUV, UNIL, Switzerland

## Abstract

Grey matter (GM) damage is a clinically relevant feature of multiple sclerosis (MS) that has been previously assessed with diffusion tensor imaging (DTI). Fractional anisotropy (FA) of the basal ganglia and thalamus might be increased in MS patients, and correlates with disability scores. Despite the established role of the striatum and thalamus in motor control, mood and cognition, the impact of DTI changes within these structures on motor and neuropsychological performance has not yet been specifically addressed in MS. We investigated DTI metrics of deep GM nuclei and their potential association with mobility and neuropsychological function. DTI metrics from 3T MRI were assessed in the caudate, putamen, and thalamus of 30 MS patients and 10 controls. Sixteen of the patients underwent neuropsychological testing. FA of the caudate and putamen was higher in MS patients compared to controls. Caudate FA correlated with Expanded Disability Status Scale score, Ambulation Index, and severity of depressive symptomatology. Putamen and thalamus FA correlated with deficits in memory tests. In contrast, cerebral white matter (WM) lesion burden showed no significant correlation with any of the disability, mobility and psychometric parameters. Our findings support evidence of FA changes in the basal ganglia in MS patients, as well as deep GM involvement in disabling features of MS, including mobility and cognitive impairment. Deep GM FA appears to be a more sensitive correlate of disability than WM lesion burden.

## Introduction

In addition to the hallmark demyelinating white matter (WM) lesions, grey matter (GM) damage in MS has been demonstrated in the early histopathological descriptions of the disease [1], [2] but only recently has been characterized with modern neuroimaging techniques. Newer MRI approaches have improved sensitivity to detect cortical [3]–[6] and subcortical [7]–[9] GM abnormalities in MS patients. These GM abnormalities can be detected at the early stages of the disease [10], [11], and are associated with physical disability and cognitive impairment [12], [13]. Volumetric MRI studies showed that among cerebral GM regions, deep grey nuclei, such as basal ganglia and thalamus, seem prominently susceptible to neurodegeneration early in the course of the disease [14], [15]. Moreover, subcortical atrophy showed stronger association with cognitive performance than whole-brain atrophy [16]. DTI, a MRI-based technique traditionally used for characterizing microstructural properties of WM tracts, has been recently employed in a few studies to detect microstructural abnormalities in the deep GM (Table 1) [17]–[23]. Increased tissue anisotropy of the basal ganglia and the thalamus have been reported in relapsing-remitting (RR) and secondary-progressive (SP) MS patients [17], [20]–[23]. The clinical relevance of these changes is supported by their association with physical disability and disease duration [23].

**Table 1:** Summary of the previous most relevant studies showing DTI abnormalities in the grey matter in MS patients.

Reference	Subjects	Regions of Interest	DTI Indices	Results
Ciccarelli et al., 2001 [17]	RRMS, SPMS, Ctrl	Caudate head, putamen, thalamus, hippocampus, cerebellar GM (9-pixel square ROIs placed bilaterally in each anatomical region)	FA, MD	FA – MS(combined)>Ctrl in the basal ganglia, putamen and thalamus MD – Ctrl>MS(combined) in the putamen
Bozzali et al., 2002 [18]	RRMS, PPMS, Ctrl	Whole GM	MD	Ctrl>RRMS>PPMS
Vrenken et al., 2006 [19]	RRMS, SPMS, PPMS, Ctrl	Whole NAGM; bilateral ROIs analysis of hippocampus, and cingulate, frontal, insular, parieto-occipital, striate cortex	FA, MD	FA – MS(combined)>Ctrl in whole NAGM, frontal, insular, parieto-occipital and striate cortex; RRMS>SPMS in parieto-occipital cortex; Ctrl>SPMS in hippocampus, frontal, insular, parieto-occipital and striate cortex MD – MS(combined)>Ctrl in all ROIs; SPMS>PPMS>RRMS in insular cortex; PPMS>RRMS in striate cortex
Hasan et al., 2009 [20]	RRMS, Ctrl	Caudate	FA, MD	FA and MD – RRMS>Ctrl
Hasan et al., 2011 [21]	CIS, RRMS, SPMS, Ctrl	Thalamus	FA, MD, AD, RD	MD – MS(combined)>Ctrl
Hasan et al., 2012 [22]	RRMS, Ctrl	Cerebral cortical GM and deep GM structures (as segmented by FreeSurfer)	FA, MD, AD, RD	FA, MD, AD, RD – RRMS>Ctrl in almost all the GM structures analyzed
Hannoun et al., 2012 [23]	RRMS, SPMS, Ctrl	Caudate, thalamus	FA, MD	FA – RRMS>Ctrl and SPMS>Ctrl in both the caudate and thalamus; SPMS>RRMS in both the caudate and thalamus MD – RRMS>Ctrl and SPMS>Ctrl in the thalamus

Abbreviations: AD – axial diffusivity, CIS – clinically isolated syndrome, Ctrl – control, FA – fractional anisotropy, GM – grey matter, MD – mean diffusivity, MS – multiple sclerosis, NAGM – normal-appearing grey matter, PPMS – primary progressive multiple sclerosis, RD – radial diffusivity, RRMS – relapsing-remitting multiple sclerosis.

Striatal nuclei and the thalamus are critical nodes of topographically organized connections between cortical and subcortical areas involved in processing motor, affective and cognitive information [24].

Here we report on DTI changes in the deep GM nuclei of MS patients. To assess whether DTI GM abnormalities have a clinical impact, we assessed associations of diffusion characteristics of the caudate, putamen and thalamus with the Expanded Disability Status Scale (EDSS) and gait (ambulation index (AI) and the Timed 25-Foot Walk (T25-FW)). In a smaller number of subjects we also performed a preliminary exploration of the relationship between DTI findings and neuropsychological status.

## Methods

### Subjects and study design

Thirty patients with MS (25 RR and 5 SP) were consecutively enrolled for this study (Table 2). Ten subjects with no neurological symptoms or known major medical disorders were recruited as controls. There was no significant difference in age and gender distribution between the controls and MS patients (Table 2). Inclusion criteria for MS patients were: 1) age 18–60; 2) diagnosis of RRMS or SPMS [25]; 3) no history of other major medical disorders; and 4) no relapse or corticosteroid use within 4 weeks before MRI examination. All MS patients underwent a neurologic examination by an MS specialist neurologist, including EDSS scoring [26], and were treated with disease-modifying therapies according to the standard of care. Detailed selection criteria for the controls have been specified elsewhere [27].

**Table 2:** Demographic, clinical and MRI characteristics.

	Control (n = 10)	MS (n = 30)	p
Age [years; mean±SD (range)]	41±9 (32–51)	43±8 (28–57)	0.41 a
Gender [F/M]	7/3	18/12	0.57 b
Disease Category [RR/SP]	-	25/5	-
Disease Duration [years; mean±SD (range)]	-	11±10 (1–35)	-
EDSS [median (range)]	-	1.5 (0–6.5)	-
Ambulation Index [median (range)]	-	1 (0–6)	-
Timed 25-Foot Walk [s; mean±SD (range)]	-	7.93±12.41 (3–61)	-
FLAIR lesion volume [ml; mean±SD (range)]	-	9.65±7.83 (2–30)	-

a Mann-Whitney test;

b Chi-squared test.

The Institutional Review Board of the Brigham and Women's Hospital (Partners Healthcare Human Research Committee) approved this study, and all subjects gave written informed consent.

### Functional Assessment

Gait performance of all MS patients was assessed using the AI [28] and the T25-FW [29]. Sixteen MS patients (13 RR and 3 SP) were administered the Minimal Assessment of Cognitive Function in MS, a test battery designed to assess the cognitive domains most commonly affected in MS [30]. They were also administered the Center for Epidemiologic Studies Depression Scale [31] to assess depressive symptoms. The Minimal Assessment of Cognitive Function in MS battery consists of seven cognitive tests yielding 11 raw scores (see Table 3). The raw scores on cognitive measures were converted into z-scores using the means and standard deviations derived from a reference group of 20 control subjects (different from the ten subjects included as control group for MRI measurements) [32]. Any z-score ≤−1.5 was considered impaired. Patients were labeled as having overall neuropsychological impairment if they demonstrated impairment on two or more tests. Center for Epidemiologic Studies Depression Scale scores ≥36 were considered to be in the depressed range. All clinical and neuropsychological assessments were performed within 3 months from the MRI scan at the Partners MS Center.

**Table 3:** The Minimal Assessment of Cognitive Function in MS battery: cognitive domains, neuropsychological tests and component scores.

Cognitive Domains	Neuropsychological Tests	Component Scores
Processing speed/Working memory	Paced Auditory Serial Addition Test	3″ Total Score
		2″ Total Score
	Symbol Digit Modalities Test	Total Score
Learning and Memory	California Verbal Learning Test-II	Total Learning
		Delayed Free Recall
	Brief Visuospatial Memory Test-Revised	Total Recall
		Delayed Free Recall
Executive Functions	Delis–Kaplan Executive Function System Free Sorting Test	Total Confirmed Correct Sorts
		Total Description Score
Visual perception/Spatial processing	Judgment of Line Orientation	Total Score
Language	Controlled Oral Word Association Test	Total Score

### MRI acquisition

Whole head images were acquired on a 3T scanner (Signa; GE Healthcare, Milwaukee, Wisconsin) using a head-only phased array coil. Scans were obtained from all subjects using a research protocol, including the following MRI sequences:

1. FLAIR: TR/TE/TI = 9000/151/2250 ms, slice thickness  = 2 mm (70 slices - no gap), matrix size  = 256×256, pixel size  = 0.976×0.976 mm, acquisition time  = 9 minutes, number of signal averages  = 1.
2. DTI: TR/TE = 13000/86 ms; slice thickness  = 3 mm (39 slices - no gap); pixel size  = 1.9×1.9 mm interpolated to 0.97×0.97 during image reconstruction on the scanner; b-factor  = 0 (5 averages) and 1000 s/mm^2^ (repeated at 21 diffusion gradient directions).

### Image Analysis

#### DTI analysis

The FMRIB Diffusion Toolbox of FSL (http://fsl.fmrib.ox.ac.uk) was used to correct for head motion and eddy current distortion. DTI volumes were then spatially aligned to a set of synthetic images generated by a first-pass analysis of the data, as detailed hereafter. Following the method of Bai and Alexander [33], an initial diffusion tensor map was created using FSL software for eddy current correction and Camino software (http://cmic.cs.ucl.ac.uk/camino) for diffusion tensor calculation. Synthetic images for each diffusion direction were generated from this initial map, and used as registration targets for the original DTI images. Each DTI image was co-registered to the corresponding synthetic image and resampled to a 1×1×1 mm matrix, using IRTK software (www.doc.ic.ac.uk/~dr/software). To limit resampling artifacts Gaussian interpolation, with the smoothing kernel full-width at half maximum equal to the acquisition matrix element size, was applied. The diffusion tensor was then estimated using Levenburg–Marquardt optimization with outlier rejection to remove artifacts such as signal loss due to cardiac pulsation using Camino software [34]. After diagonalization of the estimated tensor matrix, the fractional anisotropy (FA), mean diffusivity (MD), and axial diffusivity (AD) were calculated, using Camino.

#### Region of interest definition on DTI maps

**Figure 1:**
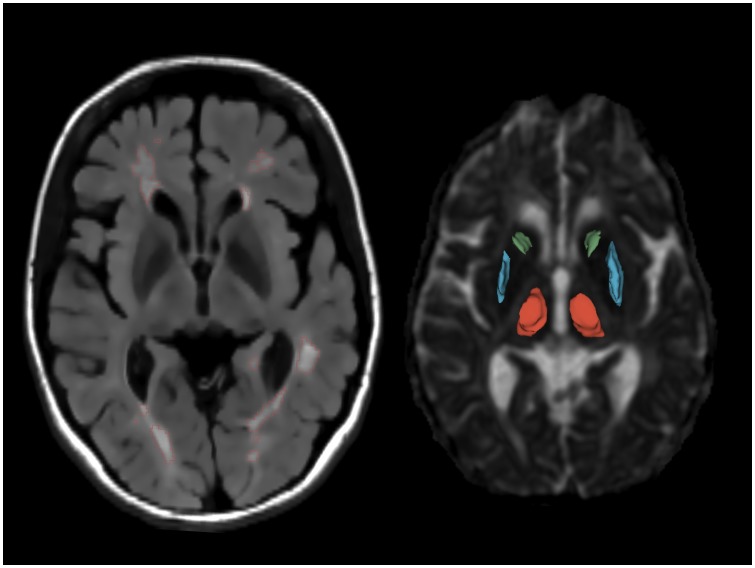
MRI example of white matter lesions and the segmented deep grey matter structures. Lesions and anatomical regions of interest are illustrated on a set of MR images from a 53 year-old male MS patient. Left – axial FLAIR; right – axial diffusivity map. The output of computer-assisted segmentation of the lesions is shown on FLAIR. The 3D models of the manually outlined caudate (green), putamen (blue) and thalamus (red) are overlaid on the fractional anisotropy map of a single section containing these structures.

The 3D extent of the caudate head, the putamen and the thalamus was manually outlined on AD maps using 3D Slicer (www.slicer.org) by two trained physicians (MC, GW) blind to demographic and clinical data (Figure 1). This approach has been previously validated by direct comparison to a reference, automated method based on co-registration of anatomical parcellation maps (obtained by applying the FreeSurfer analysis pipeline to T1-weighted images) to the DTI images [23]. Mean FA and MD of each region of interest (ROI) (left and right combined) outlined by one of the two physicians were included in the statistical analyses. For FA, intra-rater intraclass correlation coefficient (r^2^) was 0.96 for caudate, 0.94 for putamen and 0.96 for thalamus; inter-rater r^2^ was 0.93 for caudate, 0.92 for putamen and 0.92 for thalamus. To assess whether the FA values were impacted by partial volume averaging with surrounding tissue, we measured FA of the three structures of interest after one- and two-pixel erosion of the ROIs in the control group. The mean FA after one- or two-pixel erosion showed no statistically significant difference from the raw (non-eroded) values (Figure S1). Since the variance of the measured FA increased with the number of pixels eroded, we opted to use the non-eroded ROIs for our primary analysis. Dilation of the ROIs, on the other hand, did affect the FA values significantly (Figure S1). Group differences in caudate and putamen FA were demonstrated using measurements obtained from both eroded (one-pixel) or non-eroded ROIs (data not shown).

#### WM lesion volume measurement

One trained physicians (MC) supervised by an expert in image analysis (AC), both blinded to the clinical data, determined cerebral WM lesion volume on FLAIR images [15], [32] using a local-thresholding-based, semi-automated edge-finding tool, and manual correction, in Jim 5 (www.xinapse.com) (Figure 1). FLAIR images were also reviewed by two neuroimaging experts (MC, MN) to assess the presence of MS lesions in the caudate, putamen and thalamus. No subject showed lesions in the three deep gray matter structures of interest.

### Statistical analysis

Statistical analysis was performed using SPSS 13 (SPSS Inc., Chicago, Illinois). Mann-Whitney and Chi-squared tests were used to assess differences between patients and controls in age and gender, respectively. Group differences in the DTI metrics between MS patients and controls were analyzed by general linear models, including age and gender as covariates. We measured the association between quantitative MRI measures, as well as between MRI and clinical parameters by Spearman's rho. Age- and gender-adjusted partial correlations were assessed for the DTI variables that showed significant correlations with clinical parameters. Analyses involving neuropsychological test scores were also adjusted for educational status (partial correlation). Throughout the analysis the threshold of significance was ≤0.05. Due to the exploratory nature of the study, no correction was made for the number of statistical tests.

## Results

### Subject demographics and relationship with DTI metrics

Demographic and clinical characteristics of the study population are summarized in Table 2. No significant age or gender differences were found between MS patients and controls.

**Figure 2:**
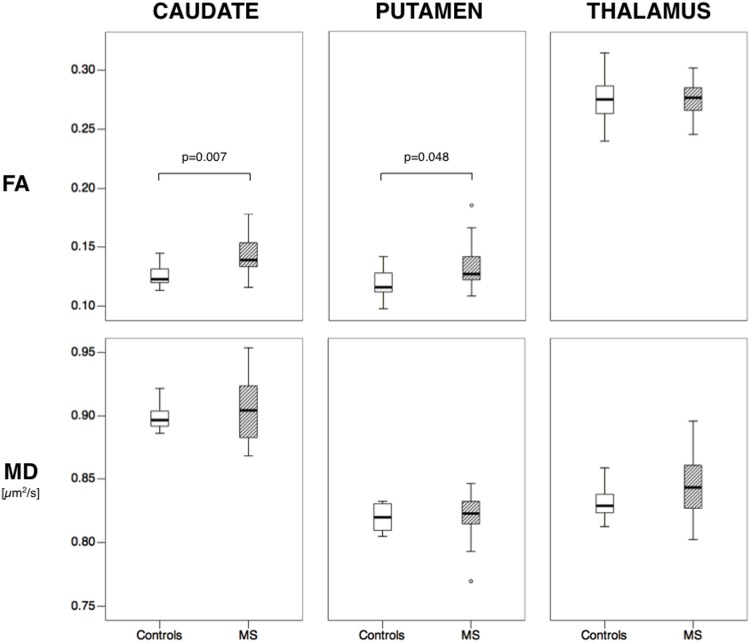
Boxplots showing the distributions of fractional anisotropy and mean diffusivity values in the caudate, putamen and thalamus in patients with multiple sclerosis (MS) and controls. The significant p-values for fractional anisotropy difference between the two groups are reported (general linear model, controlling for age and gender). Thick lines inside the boxes indicate the median value. The whiskers indicate the top and bottom quartiles. Circles are outliers. Fractional anisotropy is a scalar value between 0 and 1; Mean diffusivity is expressed in µm^2^/s.

FA values of the caudate, putamen and thalamus were normally distributed (Figure S2), and intercorrelated (p<0.02). The striatal structures showed similar FA values while the thalamus FA was higher (Table 4; Figure 2). Among the three structures of interest, the caudate showed the highest MD (Table 4; Figure 2). We found no significant differences between left and right in the FA or MD values of the three structures of interest. There was a correlation between MD values in the caudate and putamen (rho = 0.338, p = 0.035), but not between the MD of these two structures and the thalamus. Thalamic FA was higher in men than women (p = 0.043). We also found a positive correlation between both FA and MD of the thalamus and age in the whole study population (MS patients combined with controls) (rho = 0.428, p = 0.006 for FA; rho = 0.334, p = 0.038 for MD; Figure S3). For thalamic FA similar results were obtained also in the MS and control groups separately. Due to these findings, and other published evidence of age and gender influence on DTI characteristics of the deep GM [35], we controlled for age and gender throughout the statistical analyses.

**Table 4:** DTI metrics of the deep grey matter structures.

Structure/DTI metric	All subjects (n = 40)	Controls (n = 10)	RRMS+SPMS (n = 30)	RRMS (n = 25)	SPMS (n = 5)	p*
*Caudate*						
FA	0.139±0.017	0.126±0.010	0.143±0.017	0.142±0.017	0.150±0.011	0.007 (0.016)
MD	0.903±0.022	0.901±0.012	0.905±0.025	0.905±0.024	0.899±0.034	0.632 (0.628)
*Putamen*						
FA	0.130±0.017	0.120±0.013	0.132±0.017	0.132±0.015	0.138±0.028	0.048 (0.035)
MD	0.820±0.016	0.821±0.011	0.820±0.017	0.819±0.018	0.821±0.020	0.999 (0.748)
*Thalamus*						
FA	0.277±0.017	0.278±0.022	0.276±0.016	0.274±0.016	0.288±0.022	0.495 (0.456)
MD	0.841±0.022	0.831±0.013	0.844±0.022	0.840±0.020	0.864±0.022	0.134 (0.247)

Data are expressed as mean±SD.

* p-values (general linear model) refer to comparison between all MS patients and controls.

Significance p-values for comparison between RR-MS patients and controls are reported in parentheses.

Abbreviations: Fractional Anisotropy (FA), Mean Diffusivity (MD), Multiple Sclerosis (MS), Relapsing Remitting

(RR), Secondary Progressive (SP). FA is a scalar value between 0 and 1. MD is expressed in µm^2^/s.

### DTI comparisons between MS patients and controls

FA of the caudate and putamen was higher in the MS patients compared to controls (Figure 2; p = 0.007 and p = 0.048, respectively), independently of age and gender. Subgroup analysis comparing only RRMS patients and controls showed similar results (Table 4). No significant differences between patients and controls were observed in the FA of the thalamus and MD values of all three structures of interest.

### Relationship between MRI and clinical parameters in MS patients

**Figure 3:**
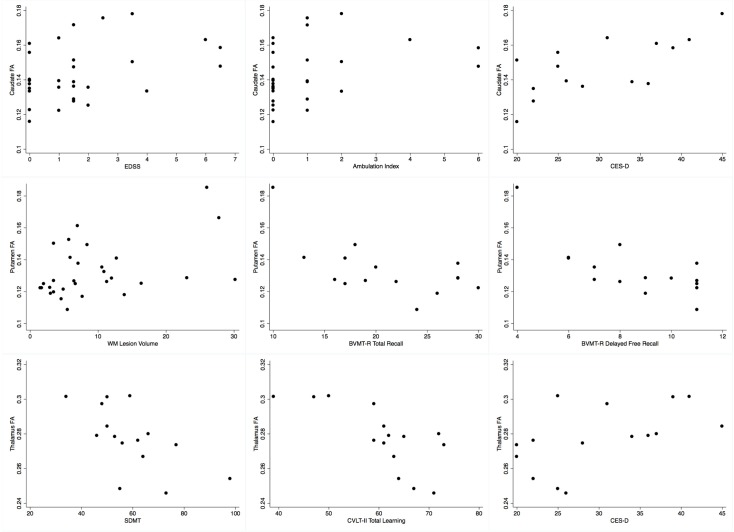
Scatterplots showing the observed significant correlations between fractional anisotropy (FA) of the three deep grey matter structures of interest and other variables of clinical relevance. Caudate FA showed a positive correlation with the Expanded Disability Status Scale (EDSS), Ambulation Index and depressive symptomatology as measured by the Center for Epidemiologic Studies Depression scale (CES-D). Putamen FA showed a positive correlation with White Matter (WM) Lesion Volume, as well as an inverse correlation with the Brief Visuospatial Memory Test-Revised (BVMT-R) Total Recall and Delayed Free Recall scores. Thalamus FA showed an inverse correlation with the Symbol Digit Modalities Test (SDMT) and the California Verbal Learning Test-II (CVLT-II) Total Learning scores, as well as a positive correlation with the CES-D score.

In MS patients caudate FA was positively correlated with EDSS (r = 0.424, p = 0.022) and AI (r = 0.388, p = 0.037), independently from age and gender (Figure 3). We found no significant correlations between EDSS or AI and DTI metrics of the putamen or thalamus. No significant association was found between the T25-FW and DTI metrics in the caudate, putamen, or thalamus.

Among the cognitively tested MS subgroup, 4 out of 16 patients had overall neuropsychological impairment, 8/16 patients showed impaired Controlled Oral Word Association Test scores, 5/16 showed impaired Delis–Kaplan Executive Function System Free Sorting Total Correct Sorts scores indicating impaired executive functions, 2/16 showed abnormal Brief Visuospatial Memory Test - Revised Total Recall scores, 1/16 showed impaired performance on the Symbol Digit Modalities Test, the Judgment of Line Orientation test or the California Verbal Learning Test (2^nd^ edition) Total Learning, none showed impaired working memory as assessed by the Paced Auditory Serial Attention Test (both the 3 and 2-second versions). In addition, 5/16 MS patients had Center for Epidemiologic Studies Depression Scale scores in the depressed range. FA was higher in MS patients with overall neuropsychological impairment compared to those without, in the putamen (p = 0.050) and thalamus (p = 0.027), but did not reach statistical significance in the caudate (p = 0.088). We found the following correlations (Table 5; Figure 3): caudate and thalamus FA with severity of depressive symptomatology; putamen and thalamus FA with cognitive deficits in memory tests; and between thalamus FA and attention deficits. After controlling for age, gender and educational status only the following associations remained significant: caudate FA and severity of depressive symptomatology (r = 0.604, p = 0.038); putamen FA and Brief Visuospatial Memory Test score (Brief Visuospatial Memory Test - Revised Delayed Free Recall, r = −0.710, p = 0.010).

**Table 5:** Spearman's correlations between fractional anisotropy of the deep grey matter nuclei and neuropsychological status in the cognitively tested MS subgroup (n = 16).

	Caudate FA *rho* (p)	Putamen FA *rho* (p)	Thalamus FA *rho* (p)
Brief Visuospatial Memory Test/Total Recall	−0.074 (0.786)	−0.545 **(0.029)**	−0.310 (0.242)
Brief Visuospatial Memory Test/Delayed Free Recall	−0.183 (0.497)	−0.690 ***(0.003)***	−0.414 (0.111)
Center for Epidemiologic Studies Depression Scale	0.716 ***(0.002)***	0.353 (0.180)	0.640 **(0.008)**
Controlled Oral Word Association Test	−0.146 (0.590)	−0.119 (0.660)	−0.480 (0.060)
California Verbal Learning Test/Total Learning	−0.191 (0.478)	−0.250 (0.350)	−0.666 **(0.005)**
California Verbal Learning Test/Long-Delayed Free Recall	0.089 (0.743)	−0.068 (0.803)	−0.290 (0.276)
Delis–Kaplan Executive Function System Free Sorting Test/Total Correct Sorts	−0.200 (0.457)	−0.405 (0.119)	−0.251 (0.349)
Delis–Kaplan Executive Function System Free Sorting Test/Total Description	−0.121 (0.655)	−0.427 (0.099)	−0.196 (0.466)
Judgment of Line Orientation	−0.070 (0.797)	−0.030 (0.913)	0.235 (0.381)
Paced Auditory Serial Addition Test	−0.148 (0.585)	0.133 (0.624)	−0.180 (0.505)
Symbol Digit Modalities Test	−0.458 (0.074)	−0.345 (0.191)	−0.620 **(0.010)**

All values shown are not corrected for age and gender with statistical significance (p≤0.05) indicated in bold. Values shown in italics are those that remained significant after correction for age, gender and educational status.

Cerebral FLAIR WM lesion burden correlated positively only with putamen FA (r = 0.397, p = 0.030; Figure 3) and disease duration (r = 0.462, p = 0.010), and did not correlate with any of the disability, mobility and neuropsychological parameters (p-values >0.1).

## Discussion

The primary aim of this study was to assess microstructural damage of the deep GM in MS patients using DTI and to explore its association with clinical and neuropsychological measures. Consistent with previous studies we found that MS patients showed increased FA values in the caudate and putamen compared to control subjects [17], [20], [23]. Increased tissue anisotropy of the basal ganglia is thought to reflect microstructural GM damage [17], [20], [23]. Interpretation of the observed DTI abnormalities in terms of their histological correlates is still debated. Specifically, the GM damage may be either secondary to inflammatory demyelination in the WM (e.g., via Wallerian degeneration) or the result of processes involving primarily the GM [10], [36]. In both cases, FA would likely increase as a consequence of damage to the GM nuclei. The association between putamen FA and WM lesion volume would support the former mechanism, i.e. a secondary, rather than a primary injury. However, the lack of association of WM lesion volume with caudate and thalamic FA does not support this hypothesis. These interpretations are based on indirect evidence and remain speculative. Previous studies have hypothesized different histopathological substrates that may explain the FA increase in the GM. Dendritic stripping, resulting in reduced barriers to diffusion of water molecules within the GM nuclei, has been proposed as possible contributor to the observed FA increase in deep GM nuclei [20], [23]. Iron accumulation has also been hypothesized as histopathological substrate of increased FA on the basis of T2-relaxometry data [20]. Future MRI-pathology correlation studies, as well as further studies using iron-sensitive MRI sequences (e.g., T2*-weighted gradient-recalled echo MRI or susceptibility-weighted imaging) are warranted to specifically address this issue. We did not find differences in the MD values of the structures of interest between patients and controls, or any correlations between MD and clinical parameters. These findings are consistent with previously reported higher sensitivity of FA compared to MD in detecting deep GM abnormalities in patients with MS [23].

As a secondary aim of this study we investigated possible associations between DTI changes in the deep GM structures and clinical parameters of physical disability and gait impairment. In a limited subset of patients, we also piloted a first exploratory analysis of the possible association between DTI findings in deep GM and neuropsychological performance. We found significant associations between tissue anisotropy in the deep GM nuclei and physical disability, gait and neuropsychological impairment in MS patients. Increased caudate FA values have previously been associated with higher disability scores [23], in line with our positive correlation between caudate FA and EDSS scores. In addition, we specifically addressed the impact of DTI changes in deep GM structures on motor performance by investigating their correlation with two measures of gait, i.e. the AI and T25-FW. In the MS group, increased caudate FA correlated with impaired gait as measured by the AI, but not with T25-FW. The relatively higher stability of the AI score in terms of test-retest and inter-rater reliability, together with the small size of the study sample may explain this apparent discrepancy in our results.

The caudate and putamen, collectively referred to as the neostriatum, represent the main input node of the basal ganglia circuitry receiving glutamatergic fibers from the sensorimotor cortex. The association between impaired motor function and DTI abnormalities in the caudate is consistent with its physiological role, while the lack of such correlation in the putamen is surprising, given its relevance within the sensorimotor loop.

Beside the traditional role of basal ganglia in motor control, it is now established that these structures are also involved in neural networks underlying affective and cognitive functions. For instance, lesions occurring along the connections between the basal nuclei and the prefrontal cortex are responsible for behavioral and cognitive disconnection syndromes [24]. We found a correlation between increased caudate FA and depressed mood as measured by a standardized self-administered scale evaluating depressive symptomatology (Center for Epidemiologic Studies Depression Scale). We also found an association between increased FA in the putamen and in the thalamus and impaired performance on neuropsychological tests assessing visuo-spatial and verbal memory, the Brief Visuospatial Memory Test and California Verbal Learning Test, respectively. Despite the established key role of the basal ganglia and thalamus in the neural networks involved in behavior and cognition, the impact of MRI abnormalities in these structures on neuropsychological dysfunction has been pointed out only recently in patients with MS, and has not yet been investigated by means of DTI targeting the deep GM. Our results are consistent with previous studies showing an association between deep GM MRI abnormalities (atrophy, T2 hypointensity) and neuropsychological findings [37]–[39]. Specifically, thalamic atrophy predicted impaired performance in tests assessing several cognitive domains, including verbal learning and memory [39]. T2 signal and volume abnormalities of the putamen have been associated with executive function and information-processing speed deficits [37], [38] rather than memory impairment. The association between putamen DTI abnormalities and impaired performance on the Brief Visuospatial Memory Test represents a novel finding that needs confirmation in larger studies.

Unlike our previously published findings in a clinically and demographically similar cohort [23], we were unable to reproduce thalamic FA differences between MS patients and controls. Given the coexistence of WM and GM within this structure, MS pathology results in competing influences on diffusion tensor signal, and it is therefore conceivable, that damaged thalami might paradoxically exhibit normal FA as a result of concomitant FA increase in damaged thalamic GM nuclei and FA decrease in damaged thalamic WM. Nevertheless, this discrepancy between our current and our previous study remains unexplained. Our study also failed to reproduce the associations of caudate and thalamus FA with WM lesion burden shown in other cohorts [23], [24], yet found a positive correlation between putamen FA and WM lesion burden. However, the association between thalamic FA and WM lesion volume is inconsistent throughout the studies [21], [23]. Demographics, disease duration, EDSS and WM lesion burden of our MS patients were very similar to those published previously [20], [23]. While the lack of difference in thalamic FA between MS patients and controls observed in our study may explain the inconsistency of our results with regards to this structure, we are unable to explain the discrepant results with regards to the caudate.

We acknowledge the small sample size as main limitation of our study. This may limit the general applicability of our findings, especially those regarding exploratory analyses of the relationship between DTI characteristics of the deep GM nuclei and neuropsychological status. In addition, most of the subjects showed scores within the normal range in the tests assessing both mood and cognition. This issue may limit the clinical relevance of our findings. Total WM lesion burden did not correlate with any of the clinical and cognitive outcome measures, likely for the same reasons. The threshold for significance applied throughout the analyses of the present study was not taking into account the number of comparisons performed.

Notwithstanding these limitations, our data support evidence of deep GM abnormalities associated with disabling features of MS, including mobility and cognitive impairment. Although larger studies are needed, our findings suggest that FA changes in deep GM provide a more sensitive correlate of motor disability than WM lesion burden.

## Supporting Information

Figure S1 **Boxplots showing the effect of erosion and dilation of the regions of interest (ROIs) on fractional anisotropy values in the control group.** Zero (0) indicates the raw, non-eroded segmentation; 1- and 2-PE indicate one- and two-pixel erosion, respectively; 1- and 2-PD indicate one- and two-pixel dilation, respectively. Statistically significant differences with respect to the raw segmentation are indicated with (*) (Kruskal-Wallis and Dunn's test for post-hoc comparisons). The mean FA after one- or two-pixel erosion showed no statistically significant difference from the raw (non-eroded) values; while dilation of the ROIs affected the caudate and putamen FA values significantly.
(PS) Click here for additional data file.

Figure S2 **Histograms showing the distribution of fractional anisotropy and mean diffusivity values in our study population.** Fractional anisotropy is a scalar value between 0 and 1; Mean diffusivity is expressed in µm^2^/s.
(PS) Click here for additional data file.

Figure S3 **Scatterplots showing the relationship between age (years) and the DTI indices of the three structures of interest.** Spearman's correlation coefficient and p-value of each correlation analysis is reported. Fractional anisotropy is a scalar value between 0 and 1; Mean diffusivity is expressed in µm^2^/s.
(PS) Click here for additional data file.

Table S1 **Tabulation of the 21 encoding directions of the diffusion tensor imaging protocol.**
(DOCX)
