# Supplementary figures and images for: Microstructural Changes in the Striatum and Their Impact on Motor and Neuropsychological Performance in Patients with Multiple Sclerosis
Source: PLoS One. 2014 Jul 21;9(7):e101199. doi: 10.1371/journal.pone.0101199 (PMC4105540; doi:10.1371/journal.pone.0101199)

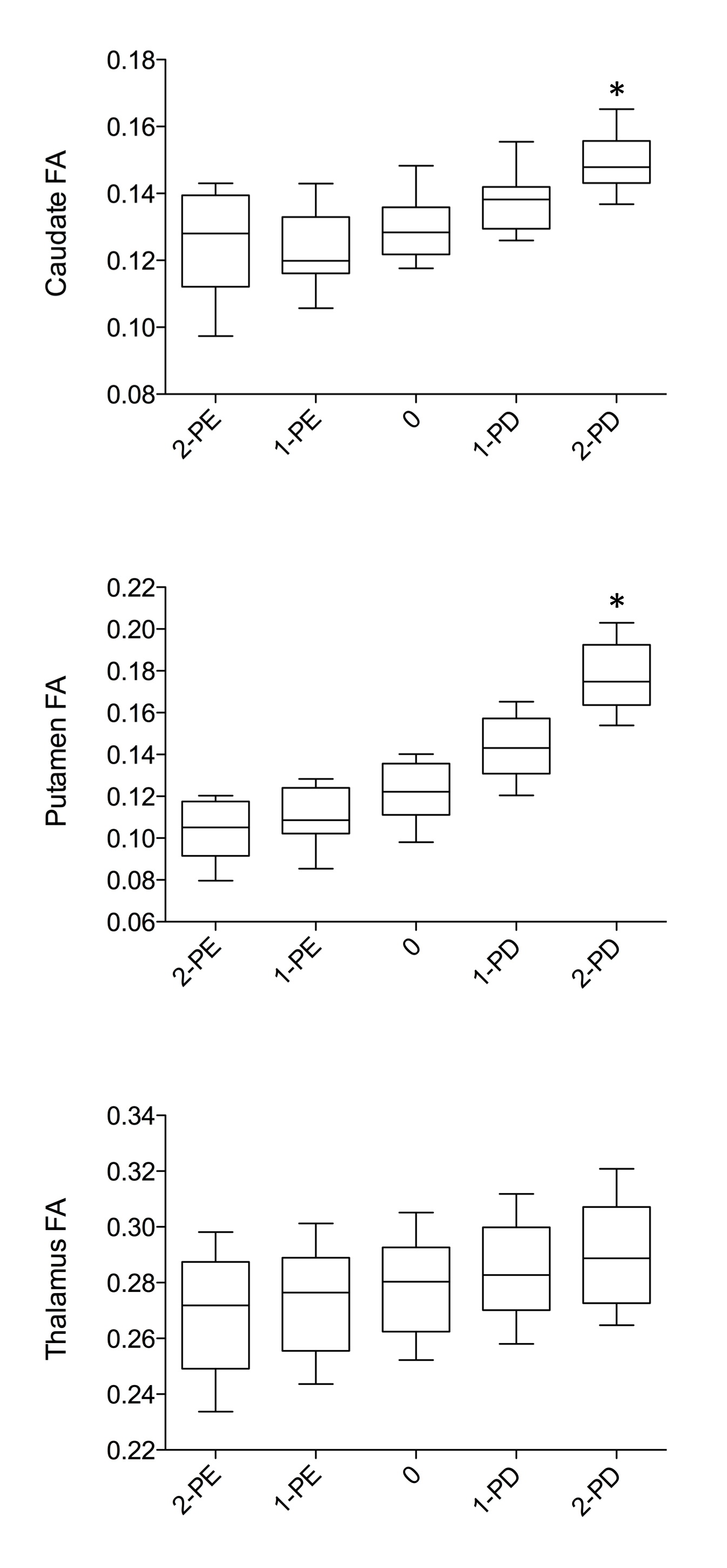

Supplement: Figure S1 — Boxplots showing the effect of erosion and dilation of the regions of interest (ROIs) on fractional anisotropy values in the control group. Zero (0) indicates the raw, non-eroded segmentation; 1- and 2-PE indicate one- and two-pixel erosion, respectively; 1- and 2-PD indicate one- and two-pixel dilation, respectively. Statistically significant differences with respect to the raw segmentation are indicated with (*) (Kruskal-Wallis and Dunn's test for post-hoc comparisons). The mean FA after one- or two-pixel erosion showed no statistically significant difference from the raw (non-eroded) values; while dilation of the ROIs affected the caudate and putamen FA values significantly. (PS) [file pone.0101199.s001.ps]

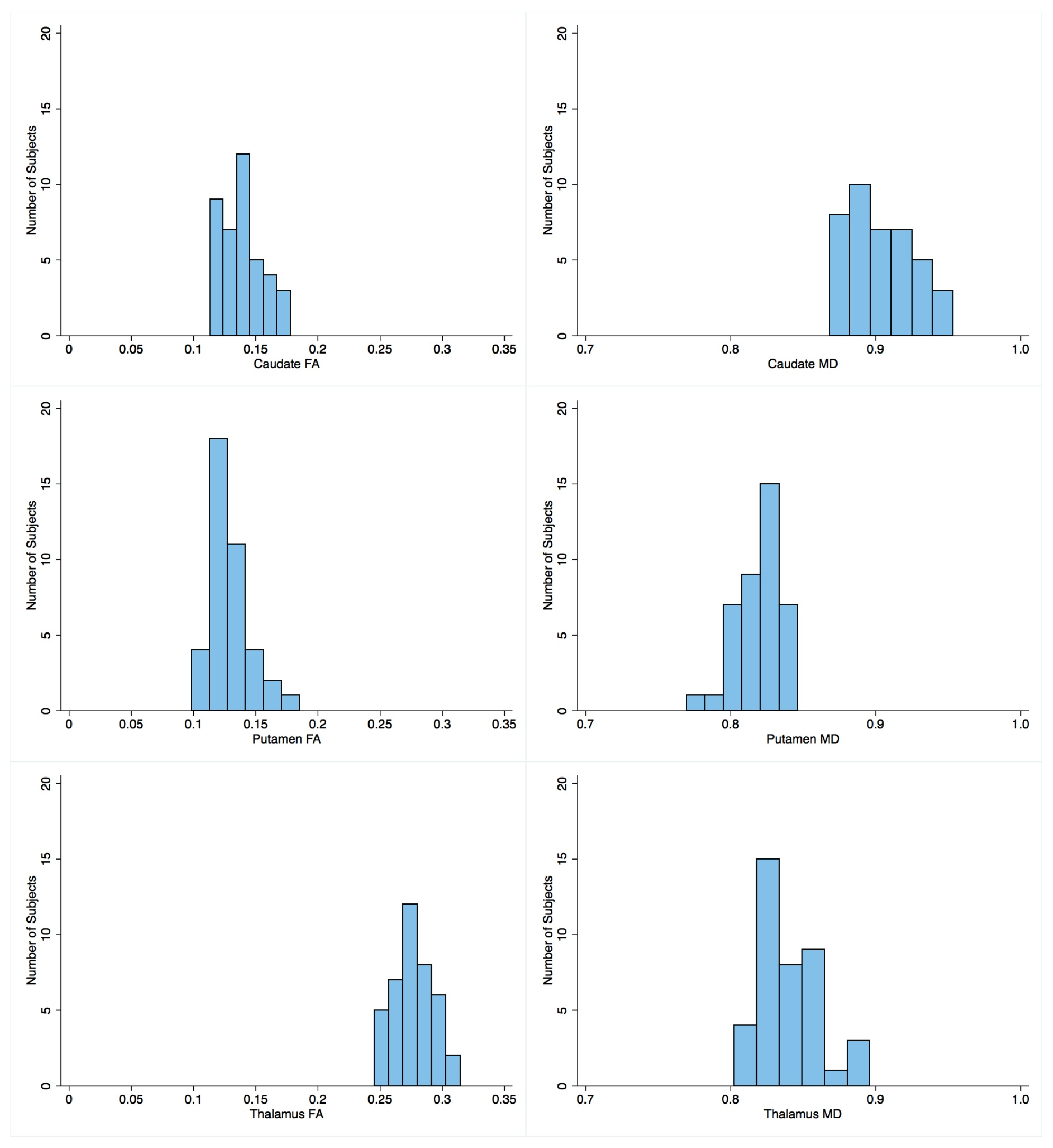

Supplement: Figure S2 — Histograms showing the distribution of fractional anisotropy and mean diffusivity values in our study population. Fractional anisotropy is a scalar value between 0 and 1; Mean diffusivity is expressed in µm2/s. (PS) [file pone.0101199.s002.ps]

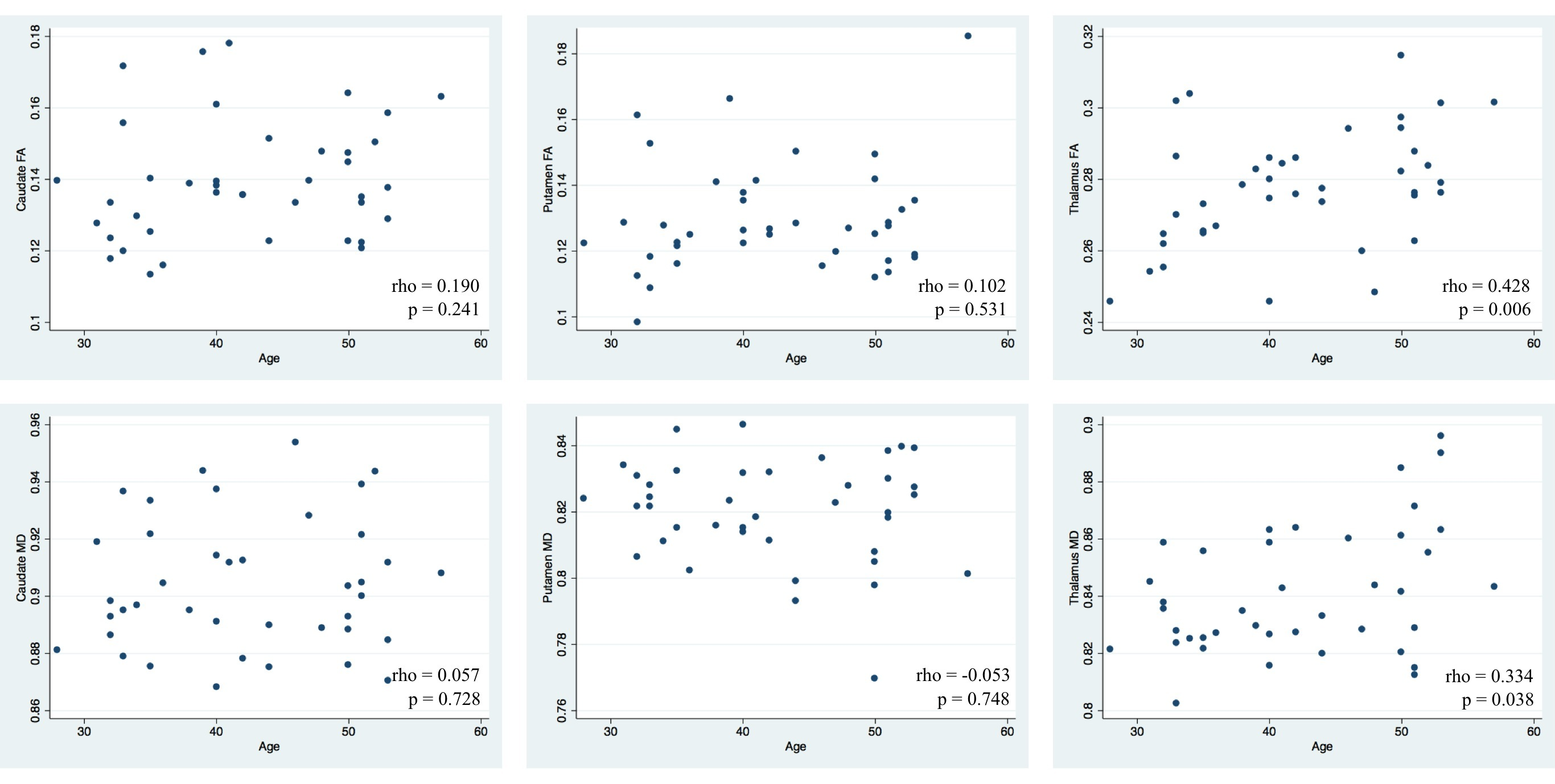

Supplement: Figure S3 — Scatterplots showing the relationship between age (years) and the DTI indices of the three structures of interest. Spearman's correlation coefficient and p-value of each correlation analysis is reported. Fractional anisotropy is a scalar value between 0 and 1; Mean diffusivity is expressed in µm2/s. (PS) [file pone.0101199.s003.ps]
