# Supplementary material for: Microstructural Changes in the Striatum and Their Impact on Motor and Neuropsychological Performance in Patients with Multiple Sclerosis
Source: PLoS One. 2014 Jul 21;9(7):e101199. doi: 10.1371/journal.pone.0101199 (PMC4105540; doi:10.1371/journal.pone.0101199)
Supplement: Table S1 — Tabulation of the 21 encoding directions of the diffusion tensor imaging protocol. (DOCX) [file pone.0101199.s004.docx]

**Table S1.** Tabulation of the 21 encoding directions of the diffusion tensor imaging protocol.

|  | ***x*** | ***y*** | ***z*** |
| --- | --- | --- | --- |
| 1 | 0.549492 | -0.499531 | -0.669721 |
| 2 | 0.229274 | 0.798245 | 0.55699 |
| 3 | -0.051585 | -0.126471 | -0.990628 |
| 4 | 0.880574 | -0.472012 | 0.042366 |
| 5 | -0.874734 | 0.472842 | -0.106114 |
| 6 | -0.464585 | 0.484633 | -0.741142 |
| 7 | -0.517653 | 0.668753 | 0.533671 |
| 8 | 0.214763 | 0.160054 | 0.963462 |
| 9 | -0.178941 | -0.7843 | -0.594014 |
| 10 | 0.517841 | -0.48367 | 0.705623 |
| 11 | 0.594929 | 0.796922 | -0.104762 |
| 12 | -0.586168 | -0.809073 | 0.042519 |
| 13 | -0.211592 | -0.690879 | 0.691314 |
| 14 | -0.180353 | 0.967957 | -0.174732 |
| 15 | -0.511468 | -0.015519 | 0.859162 |
| 16 | 0.318668 | 0.525872 | -0.788613 |
| 17 | 0.893219 | 0.152007 | -0.423147 |
| 18 | 0.254924 | -0.965695 | 0.049467 |
| 19 | 0.864508 | 0.24546 | 0.438607 |
| 20 | -0.946615 | -0.182258 | 0.2659 |
| 21 | -0.793779 | -0.243314 | -0.557416 |
